# Influence of Physical Activity Levels and Functional Capacity on Brain β-Amyloid Deposition in Older Women

**DOI:** 10.3389/fnagi.2021.697528

**Published:** 2021-07-09

**Authors:** Raquel Pedrero-Chamizo, Cassandra Szoeke, Lorraine Dennerstein, Stephen Campbell

**Affiliations:** ^1^Department of Health and Human Performance, ImFINE Research Group, Universidad Politécnica de Madrid, Madrid, Spain; ^2^Exercise and Health Spanish Research Net (EXERNET), Zaragoza, Spain; ^3^Department of Medicine, Faculty of Medicine Dentistry and Health Sciences, Centre for Medical Research (Royal Melbourne Hospital), The University of Melbourne, Parkville, VIC, Australia; ^4^Australian Healthy Ageing Organisation, Parkville, VIC, Australia; ^5^Department of Psychiatry, University of Melbourne, Melbourne, VIC, Australia; ^6^Melbourne Health, Royal Melbourne Hospital, Parkville, VIC, Australia

**Keywords:** function, cognition, Alzheimer's disease, physical activity, women, PET, APOE ε4, healthy ageing

## Abstract

Physical activity (PA) and Alzheimer's disease are associated. However, how PA influences the cerebral β-amyloid (Aβ) burden remains unclear. The aim of this study was to determine if PA levels and/or functional capacity (FC) are associated with Aβ plaque deposition, and whether these associations differed according to APOE-ε4 genotype. A total of 117 women (69.7 ± 2.6 years; 33.3% APOE-ε4-carriers) from the Women's Healthy Ageing Project cohort (WHAP) were analyzed. PA was measured using the International Physical Activity Questionnaire and, FC was evaluated using the Timed Up and Go test (TUGt). Positron emission tomography with F-18 Florbetaben was carried out to assess cerebral Aβ burden, and quantified using standardized uptake value rations. The sample was split into PA and TUGt tertiles (T1, T2 and T3), and compared according to APOE-ε4 genotype (positive/negative). There were no significant differences in Aβ accumulation according to PA tertiles and APOE-ε4 genotype. Regarding FC, APOE-ε4+ participants in the first TUGt tertile (high performance) obtained significant lower Aβ accumulations compared with the other two tertiles (*p* < 0.05). Comparing between genotypes, greater Aβ depositions were found between T2 and T3 in APOE-ε4+ compared with those who were APOE-ε4– (*p* < 0.05). Values of TUGt ≥ 6.5 s (APOE-ε4+) and 8.5 s (APOE-ε4–) were associated with an increased risk of having higher Aβ retention. In conclusion, low performance in TUGt is associated with a negative effect on brain pathology with increasing cerebral Aβ depositions in older women who are APOE-ε4+. In physically active older women (> 600 METs·min/week), higher PA levels are not associated with reduction in Aβ depositions.

## Introduction

The accumulation of amyloid beta (Aβ) peptides in the brain is recognized as the earliest detectable pathophysiological abnormality in Alzheimer's disease (AD) (Gandy, 2005). Previous studies have observed how cognitively healthy people, with a predisposition to accumulate Aβ brain, can begin to experience progressive increases in the retention of this peptide even 20 years before reaching the thresholds for amyloidosis (Rowe et al., 2010; Perani et al., 2014).

Among principal non-modifiable AD risk factors, age is the most well-known factor, showing an almost exponential increase with advancing age, especially in female sex, following by family history and genetics (e.g., APOE-ε4 carriers) (Livingston et al., 2020). However, in the absence of disease-modifying therapies for AD, there is a necessity to identify modifiable risk factors that may delay and even prevent disease.

Increasing regular exercise is considered a protective component against cognitive decline and AD (Rabin et al., 2019; Livingston et al., 2020), and is now recommended in the WHO report (WHO, 2019) on preventing dementia, since it improves cerebral perfusion, reduces neuronal loss, improves brain plasticity and preserves brain volume (Valenzuela et al., 2020), besides being an independent factor of cardiovascular health (Rabin et al., 2019). Nevertheless, there are limitations in assessing physical activity (PA) using objective methods, which often do not reflect personal physical performance. In this sense, evaluation of functional capacity (FC) has been identified as an objective measure which is a reflection of individual fitness, positively associated with many indices of health, and as early predictive factor of brain deterioration (Erickson et al., 2014; Rabin et al., 2019). However, information on the associations between PA and FC with biomarkers of AD has not been consistently replicated, particularly regarding Aβ brain deposition. The objective of this study was to determine if PA levels and/or FC are associated with Aβ plaque deposition in older women, and whether these associations differed according to APOE-ε4 genotype.

## Methods

### Sample

All data for the present study were collected from theWHAP cohort. The complete methodology of the WHAP has been described elsewhere (Szoeke et al., 2016). In brief, WHAP started in 1992, when 438 Australian women (aged between 45 and 55 years) were selected by random population sampling and enrolled into a prospective longitudinal follow up study. In the present study, participants who completed brain MRI scan plus Positron Emission Tomography (PET), completed a PA questionnaire and carried out a FC test in 2012 data collection were eligible for inclusion in the analysis (see Supplementary Figure 1). To make maximum use of the data, all valid data on PA and FC were included in this report. Consequently, sample sizes vary for the PA analysis (*n* = 117) and FC analysis (*n* = 98).

The study protocol for the WHAP project was approved by the University of Melbourne Human Research Ethics Committee and fully compliant with the guidelines of the National Health and Medical Research Council ethical standards (HREC 931149X, 1034765, 110525, 1339373, 010411, 1647448 & 1750632) and was carried out in accordance with the Declaration of Helsinki. All subjects provided written informed consent before participation.

### Cerebral Imaging

Data from PET scan were conducted with participants receiving 250 MBq of F-18 Florbetaben intravenously, with a 20 min acquisition commencing 90 min post injection. Standardized Uptake Values (SUV) were calculated for all brain regions examined and SUV ratios (SUVRs) generated by normalizing regional SUV using the cerebellar cortex. Neocortical SUVRs, a global measure of Aβ burden, is expressed as the average SUVRs of the area-weighted mean of frontal, superior parietal, lateral temporal, lateral occipital and anterior and posterior cingulate regions (Szoeke et al., 2016). A SUVRs threshold of 1.2 or greater was used to discriminate participants with an intermediate brain Aβ load (+SUVRs). Similar threshold value was identified in previous studies (Ciarmiello et al., 2019; Kim et al., 2020).

### Physical Activity and Functional Capacity

Data on PA were collected using the International Physical Activity Questionnaire (IPAQ) (Craig et al., 2003). The reported minutes per week in four activity domains (work-related PA, transportation PA, domestic PA, and recreational PA) were multiplied by the metabolic equivalent (MET) score (METs·min/week), based on the intensity of the activity being undertaken.

Data on FC were evaluated using the Timed Up and Go test (TUGt) (Podsiadlo and Richardson, 1991). The TUGt measures the time (in seconds) taken to rise from a seated position, walk 3 meters from the chair, walk back to the chair and sit down again. A shorter time to complete this test reflects better physical performance.

In the current study, participants were separated into tertiles (T1, T2 and T3) dependent upon IPAQ and TUGt based on the calculated scores.

### Confounders

Potential confounders were selected from the literature and included age, body mass index (BMI; kg/m^2^), educational level (> 12 years), physical activity, cognitive status (assessed using Mini-Mental State Examination [MMSE]) (Folstein et al., 1975), cardiovascular disease risk (assessed using Framingham score for cardiovascular risk) (Kannel et al., 1976), and APOE-ε4 genotype (positive [APOE-ε4+] or negative [APOE-ε4–]). All analyses presented are adjusted for these covariates.

### Statistical Analysis

All statistical analyses were conducted using statistical package for the social sciences (SPSS) version 24.0 (SPSS Inc., Chicago, IL, USA). The normal distribution of the variables was examined with the Kolmogorov-Smirnov test. Since SUVRs variable was not normally distributed, non-parametric statistic was utilized. Spearman correlation coefficient was used to examine associations among IPAQ, TUGt, APOE-ε4 genotype, and SUVRs. Differences in the mean SUVRs across IPAQ tertiles and TUGt tertiles were analyzed using Kruskal-Wallis test and Mann-Whitney U-test. A receiver operating characteristic (ROC) curve was generated and the Youden index was used to identify optimal cut-off points which were related with an increased risk of suffering +SUVR (SUVRs ≥ 1.2). Due to the fact that APOE-ε4 genotype shows an interaction with Aβ levels, this analysis was carried out separately obtaining two differences cut-off points according to APOE-ε4 genotype. Binary logistic regression analyses were performed to examine associations between TUGt cut-off and different thresholds for amyloid positivity, according to previous studies (Villemagne et al., 2011; Duara et al., 2019). Odds ratios with 95% confidence intervals (CI) are reported for the studying models. Model I included the independent variable. Model II incorporated all confounders. Statistical significance was set at *p* < 0.05.

## Results

### Sample Characteristics

The study participants had a mean age of 69.7 ± 2.6 years and the 33.3% of the sample was APOE-ε4+. Participant characteristics are displayed in Table 1. SUVRs correlated significantly with APOE-ε4 genotype (*Spearman's rho* = 0.234, *p* < 0.05) and TUGt values (*Spearman's rho* = *0.255, p* < 0.05).

**Table 1:** Participant characteristics.

	**Overall (*n* = 117)**
Age at testing (years)	69.7 ± 2.6
APOE ε4 carrier n (%)	39 (33.3%)
Body mass index (kg/m^2^)	28.3 ± 5.5
Energy expenditure (METs·min/week)^‡^	4070 (2129 – 7332)
TUG test (seconds)^†^	7.6 ± 1.7
MMSE (score)	28.5 ± 1.4
Past smokers n (%)	45 (38.5)
Current smoker n (%)	10 (8.5%)
Alcohol intake (≤ 2 drinks/week)	46 (39.3%)
Education (≤ 12 years) n (%)	65 (55.6%)

*APOE, apolipoprotein E; METs, metabolic equivalent score; MMSE, Mini Mental State Exam; TUG, Timed up and go*.

*Values are expressed as mean ± standard deviation unless otherwise indicated*.

‡ *Value is expressed as median (interquartile range)*.

† *TUG values on a sample of 98 subjects*.

### Association Between PA and Aβ Brain Concentrations

The sample was split into tertiles according to IPAQ scores. The first tertile (T1) was composed of subjects who reported the lowest activity (IPAQ <2769 METs·min/week) while subjects reporting the highest activity were allocated to T3 (IPAQ > 6492 METs·min/week). All subjects, except for one person, met the current PA guidelines (≥ 600 METs·min/week) as per the IPAQ and WHO (Craig et al., 2003; Bull et al., 2020). There were no statistical differences according to age, MMSE and BMI, neither among tertiles nor between APOE-ε4 genotype (*p* > 0.05).

Analyzing SUVRs between tertiles, we can observe similar values between them (*p* > 0.05). When the sample was compared taking into account the APOE-ε4 genotype, again no significant differences were observed in Aβ brain concentrations. In the same way, comparing SUVRs between APOE-ε4 genotypes (positive vs. negative) in each tertile, no statistically significant differences were found except for APOE-ε4+ participants placed in T1 who showed a tendency to accumulate greater cerebral Aβ deposits compared to APOE-ε4– allocated in the same tertile (*p* < 0.1) (Supplementary Figure 2).

### Association Between FC and Aβ Brain Concentrations

The sample was split into tertiles according to TUGt values. Individuals placed in T1 performed TUGt in lesser time than subjects allocated to T3, showing a better performance of T1 with respect to T3 (T1: TUGt <7.0 s; T3: TUGt > 7.99 s). The 23.5% of the sample obtained values for the TUGt higher than the mean of the reference values published for healthy women over 60 years old (TUGt > 8.87 s) (Long et al., 2019), denoting that the majority of the cohort (76.5%) maintains an optimal functional mobility, especially participants allocated in the first tertile.

**Figure 1:**
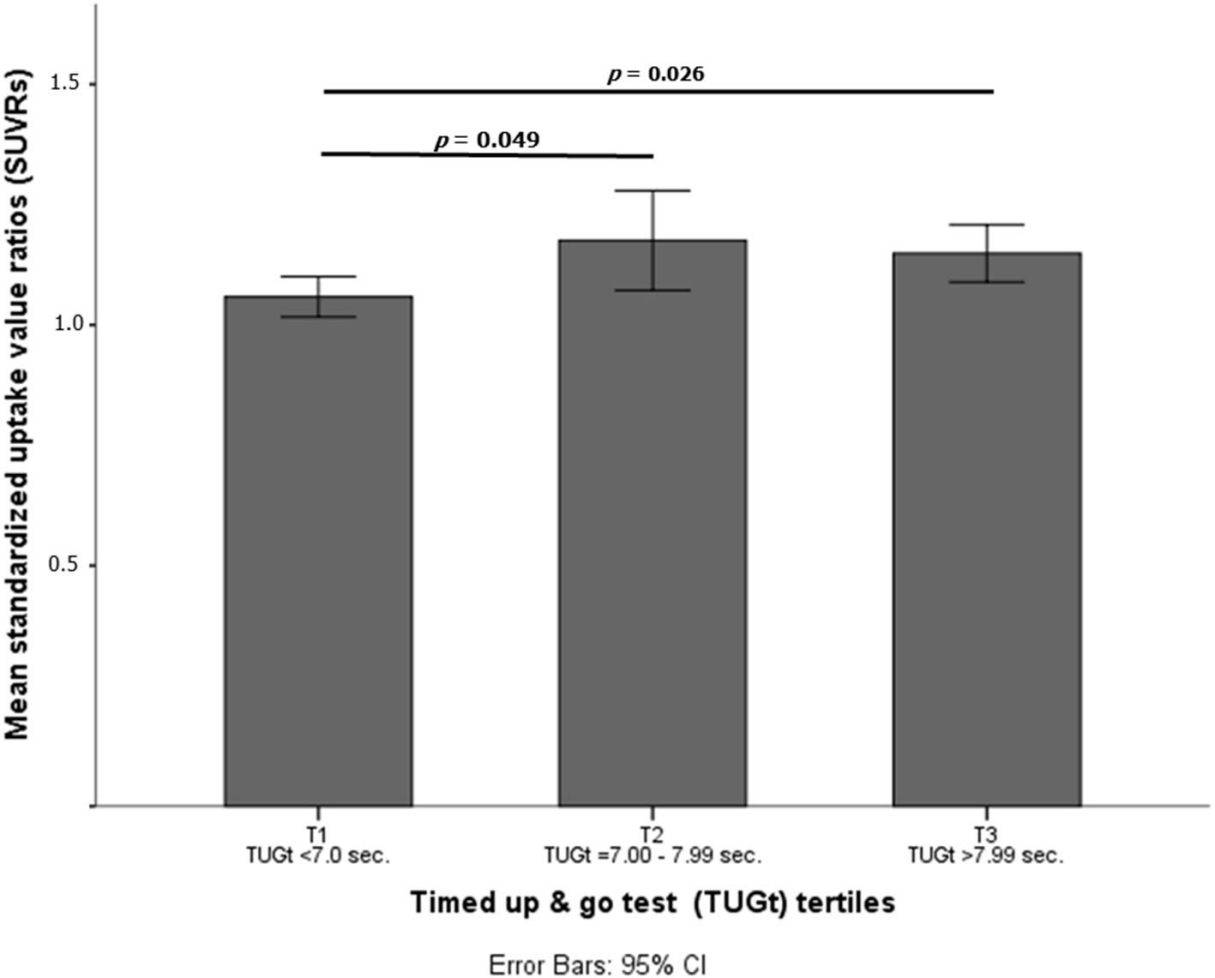
Mean standardized uptake value ratios (SUVRs) according to timed up and go test (TUGt) tertiles.

**Figure 2:**
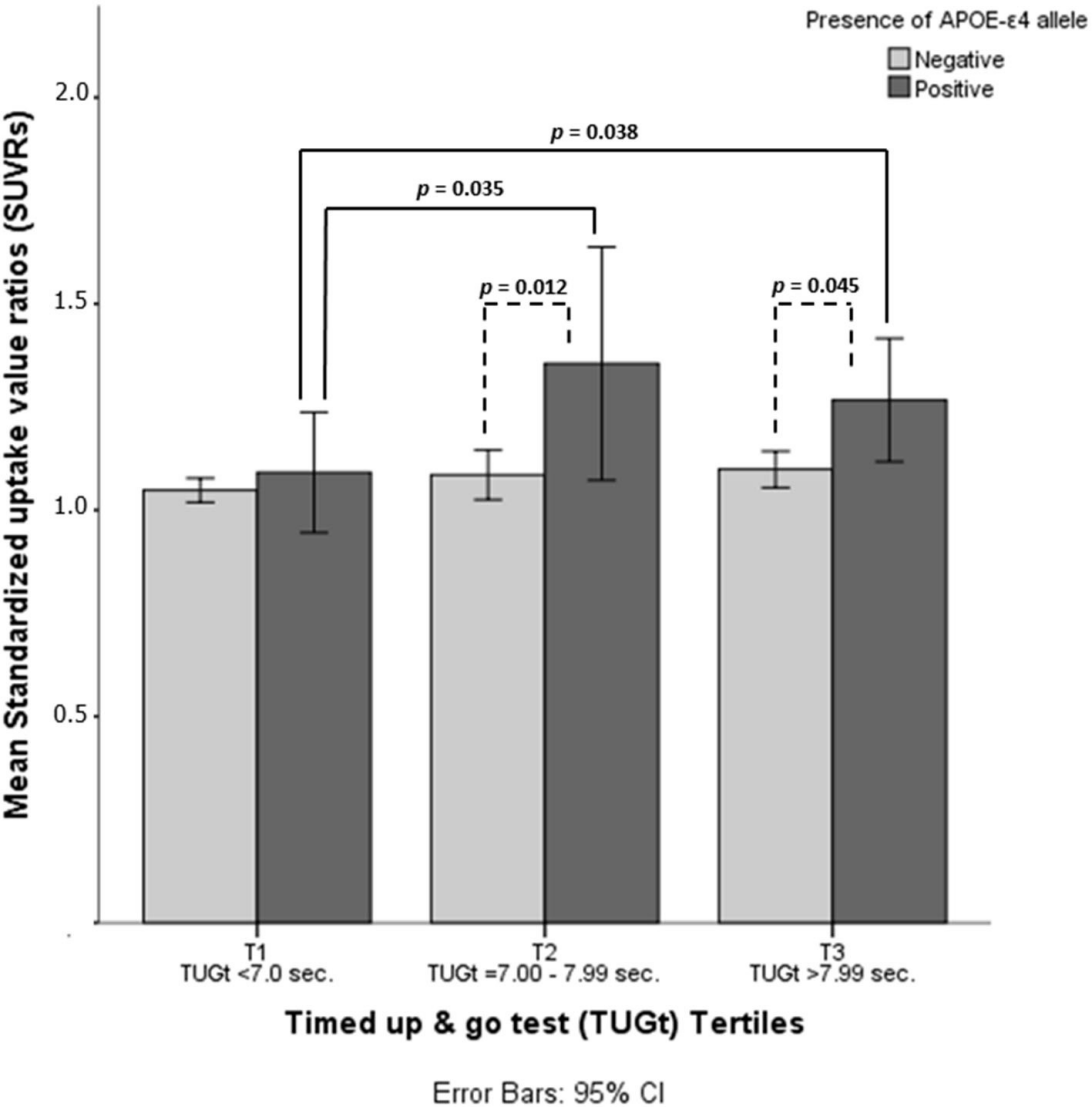
Mean standardized uptake value ratios (SUVRs) according to timed up and go test (TUGt) tertiles and APOE-ε4 genotypes.

Statistically significant differences were observed in brain Aβ deposits among tertiles (*p* = 0.016, see Figure 1). When the cohort was split according to TUGt tertiles and APOE-ε4 genotypes (Figure 2), APOE-ε4– participants obtained similar brain concentrations of Aβ among tertiles, except for subjects in T3 who showed a trend to accumulate greater cerebral Aβ deposits compared to T1 (*p* = 0.97) (data not shown). Regarding to APOE-ε4+, participants placed in T1 accumulated significantly lower Aβ levels compared with the other two tertiles (*p* < 0.05 in both cases). When SUVRs were compared between APOE-ε4 genotypes and according to TUGt tertiles, APOE-ε4+ subjects placed in T2 and T3 obtained significantly higher Aβ brain concentrations compared with APOE-ε4–subjects (*p* < 0.05), as shows in Figure 2.

ROC curve analysis demonstrates an area under the curve (AUC) of 0.621 and 0.644 (APOE-ε4+ and APOE-ε4–, respectively). The Youden index identified optimal cut-off values; a threshold of 6.5 s (APOE-ε4+) and 8.5 s (APOE-ε4–) maximizes sensitivity and specificity of the TUGt performance as a tool for discriminating participants with an intermediate brain Aβ load (+SUVRs ≥ 1.2). A binary logistic regression examining the association between TUGt cut-off points and +SUVR was carried out. Participants whose TUGt performance was slower than cut-off points were associated with 6.0-fold higher odds (95%*CI* = 1.962–18.644) for having +SUVRs compared with those performing TUGt faster than cut-off points (Table 2). The adjustment for confounders (Model II) did not significantly change this result. In addition, a new binary logistic regression was run to analysis the association between TUGt cut-off points proposed and amyloidosis cut-off points reported by other authors (Villemagne et al., 2011; Duara et al., 2019) obtaining similar results (see Table 2).

**Table 2:** Binary logistic regression examining the association between TUGt cut-off points and different thresholds for amyloid positivity.

**Amyloid positive thresholds**		**Risk values of TUGtδ!!break OR (95% CI)**	***p* value**
Cut-off ≥ 1.20 SUVRs	Model I	6.05 (1.96 – 18.64)	0.002
	Model II	8.12 (2.26 – 29.19)	0.001
Cut-off ≥ 1.31 SUVRs^†^	Model I	4.12 (1.17 – 14.53)	0.027
	Model II	4.97 (1.22 – 20.22)	0.025
Cut-off ≥ 1.40 SUVRs^‡^	Model I	7.35 (1.47 – 36.80)	0.015
	Model II	17.21 (2.12 – 139.86)	0.008
Cut-off ≥ 1.42 SUVRs^†^	Model I	6.23 (1.22 – 31.82)	0.028
	Model II	17.21 (2.12 – 139.86)	0.008

*CI, confident interval; OR, odds ratio; SUVRs, standardized uptake values ratios; TUGt, timed up and go test*.

† *Amyloid positivity threshold by Duara et al., 2019;*

‡ *Amyloid positivity threshold by Villemagne et al., 2011. δTUGt cut-off points: ≥ 6.5 s (APOE-ε4+) and ≥ 8.5 s (APOE-ε4–)*.

*Model I, included the independent TUGt cut-off variable; Model II, adjusted by age, BMI, physical activity (IPAQ score), education (>12 years), cognitive status (MMSE score), and cardiovascular disease risk (Framingham score)*.

## Discussion

This study evaluated associations between PA (measured by IPAQ) and FC (measured by TUGt) and cerebral Aβ deposition (assessed using F-18 florbetaben) in older women from the WHAP cohort. The main findings of the present study are: (1) in physically active older women (> 600 METs·min/week), higher PA levels are not associated with reduction in cerebral Aβ deposition; (2) better performance in the TUGt is associated with lower Aβ brain deposition in APOE-ε4+; (3) a high PA level and faster TUGt results appear to lead to less cerebral Aβ retention in APOE-ε4+; (4) TUGt results slower than 6.5 (APOE-ε4+) and 8.5 s (APOE-ε4–) are associated with an increased risk of amyloid accumulation at levels above the accepted normal range.

Physical inactivity has been identified as a risk factor on various chronic diseases and has been associated with a higher risk of mortality (Kyu et al., 2016; Ekelund et al., 2020), while an active lifestyle has been associated with numerous benefits. Regarding AD-biomarkers, few studies have evaluated the potential benefits of habitual PA on Aβ brain concentrations not finding a significant association between cerebral Aβ concentrations and PA in the majority of studies (Landau et al., 2012; Okonkwo et al., 2014; Wirth et al., 2014; Schultz et al., 2015; Frederiksen et al., 2019). Nevertheless, some factors such as APOE-ε4 genotype can influence this association and not all studies took this into account. Brown et al. (2013) evaluated the association between PA and amyloid brain depositions in a cohort of 116 older people and did not find significant associations when the cohort was analysis on the whole; however, when the sample was stratified by genotypes, APOE-ε4+ participants with more active lifestyle showed significant lower cerebral Aβ concentrations than their counterparts. Conversely, our findings did not find associations between PA levels and cerebral Aβ accumulations, not even when the sample was split into APOE-ε4 genotypes, corroborating results presented in other observational studies (Vemuri et al., 2012; de Souto Barreto et al., 2015).

Another important factor to take into account is the PA level from which the individual starts. Previous studies (Kyu et al., 2016; Ekelund et al., 2020) have observed that the greatest health benefits are obtained by people whose activity level is greater than the 600 METs·min/week recommended by the WHO (Bull et al., 2020). In relation to cerebral Aβ concentrations, similar results were reported. Liang et al. (2010) evaluated the association of exercise on AD-biomarkers concluding that individuals who satisfied the American Heart Association's physical exercise recommendation for older adults (≥ 7.5 METs-h/week) (Nelson et al., 2007) accumulated lower cerebral Aβ concentrations compared with physically inactive elderly. Similar results were found in other studies (Head et al., 2012; Okonkwo et al., 2014) among which we can highlight the study by Head et al. (2012), in which a greater effect of exercise was observed on brain concentrations of Aβ in APOE-ε4+ physically inactive participants. When analyzing our results, we observed that the entire sample was physically active. This fact could explain the lack of statistical significance between the variables, since the main differences in relation to Aβ retention have been observed comparing people who meet the PA recommendations vs. people who do not meet them. Moreover, previous studies have established that the most health gains occur at relatively lower levels of activity (> 600 METs·min/week) up to 3000 METs·min/week, approximately (Kyu et al., 2016; Ekelund et al., 2020). In our case, participants performed a habitual PA equal to or even higher than these thresholds, with the exception of one woman, elucidating that it is a cohort with a very active lifestyle and, though these PA cut-off points are not specific for brain amyloidosis risk, it may be influenced in the same way.

Although PA and FC are related, they are separate physiological and behavioral measures that can explain different aspects and predict various health outcomes. Physical activity has been defined as any bodily movement that results in energy expenditure (Caspersen et al., 1985) while FC reflects the ability to perform activities of daily living (Lawton and Brody, 1969). In this sense, FC requires integrated efforts of the cardiopulmonary and skeletal muscle systems, providing important diagnostic and prognostic information in clinical and research settings (Arena et al., 2007), especially when it is assessment by physical tests as objective measure of physical capacity instead of questionnaires.

The most common test to assess FC in relation to brain Aβ load is gait speed. Slow walking speed has been considered a predictor of cognitive decline, dementia, disability, neuropathologies, and even death (Abellan van Kan et al., 2009; Wennberg et al., 2017). Few human studies have examined the association between cerebral Aβ load and gait speed and functional mobility, finding in most studies a stronger association between greater cerebral Aβ and lower extremity motor decline and poorer performance on multiple gait parameters among people without dementia (Nadkarni et al., 2017; Tian et al., 2017; Wennberg et al., 2017).

In this study FC was evaluated through the TUGt. It has a high correlation with other validated tests that measure pure gait speed (Bohannon, 2006), and it is appropriated for evaluating functional mobility and dynamic balance. According to our results, the TUGt evaluation showed very interesting findings with slower TUGt performance associated with greater cerebral Aβ depositions in APOE-ε4+. In addition, a positive trend was observed when comparing T1 and T3 in APOE-ε4–; however, this association did not reach statistical significance. In extension of these findings, the results of our study further affirm that cognitively healthy women who preserve a good reserve of functional mobility are associated with optimal levels of brain Aβ, especially for those with an APOE-ε4 risk genotype. Nevertheless, our findings are not supported by all authors. Dao et al. (2019) found that Aβ deposition was not associated with the TUGt. However, this study assessed a relatively small sample, in addition to not stratifying by sex and APOE genotype, both APOE-ε4+ and women being strongly associated with cerebral Aβ retention and functional mobility (Wennberg et al., 2017; Szoeke et al., 2019).

An interesting observation from our results is that women obtaining higher performance in TUGt (T1) maintain lower cerebral Aβ concentrations, regardless of APOE-ε4 genotype. In contrast, slower results in TUGt were associated with greater cerebral Aβ depositions in APOE-ε4+ participants, who had a significantly greater amyloid accrual compared to APOE-ε4– women placed in the same tertile (T2 and T3). Previous studies in this area have observed that those with the APOE-ε4+ genotype tend to accumulate higher amyloid loads than APOE-ε4–, at all ages and at all cognitive impairment levels (Duara et al., 2019). Our data suggest higher performance on functional mobility and high PA levels attenuate the negative effect of APOE-ε4+ on amyloid concentrations, slowing down brain Aβ accumulations in accordance with previous studies (Head et al., 2012).

A novel aspect of our research is that we describe cut-off points for TUGt which are related with an increase in the risk of presenting an intermediate brain Aβ load (+SUVRs ≥ 1.2). We decided to use a lower cut-off point than current thresholds for amyloid positivity with the purposes of early diagnosis and thus to afford opportunity for successful therapeutic interventions aimed at delaying disease onset and limiting further neuronal damage. To our knowledge, this is the first time that cut-off points have been established for TUGt in relation to the risk of suffering from amyloidosis. Our results indicate that slower values at the established cut-off points are associated with a greater risk of having high brain concentrations of amyloid.

Given the strong relationship between functional mobility and cognition seen in this study also relates to measures of brain pathology, other measures of FC and physical fitness known to relate to later life frailty may be useful clinical tools to help identify those at risk dementia. In this sense, TUGt is an accessible and easy measurement that may be used in clinical assessment. Further research could examine the specificity of these measures for the later life development of dementia. It will be necessary to have longitudinal studies to establish the precise association between FC and Aβ brain concentrations and the interaction with APOE-ε4 genotypes. Given we now know that amyloid accumulates over 30 years, it will be essential to examine PA over the prodrome of disease to understand if there are particular therapeutic windows or at risk periods which are relevant to proposed intervention.

We acknowledge this study had limitations. Whilst the optimal questionnaires to evaluate PA was utilized, objective measurement, such as accelerometer, could provide actual measures of activity, although the duration of this would be limited and there is evidence that when wearing the device, participants change their behavior. The relatively modest number of subjects included in the study is critical to the interpretation and generalizability of the findings. Our significant findings in a sample size around a hundred healthy women indicate that the strength of the effect is large.

Finally, as we have only one measure of amyloid accumulation we cannot address causality of these associations. Further longitudinal studies are needed to determine whether FC level predicts Aβ brain depositions, and human intervention studies are required to observe if increasing exercise influences Aβ.

In conclusion, this study provides valuable information on the association between PA and FC on AD risk. Our findings suggest that PA levels higher than global recommendations and a better performance on TUGt could be associated with slowing down cerebral Aβ retention in APOE-ε4 carriers and with a protective effect against brain Aβ depositions. Finally, TUGt performance slower than cut-off points proposed increases the risk of presenting an intermediate brain Aβ load (SUVRs ≥ 1.2).

## Data Availability Statement

The datasets presented in this article are not publicly available because of patient confidentiality and participant privacy reasons. Requests to access the datasets can be found in online repository. The name of the repository is Women's Healthy Ageing Project University of Melbourne VIC and applications for data access can bemade through the following URL: https://www.biogrid.org.au/data-directory.

## Ethics Statement

The studies involving human participants were reviewed and approved by the University of Melbourne Human Research Ethics Committee and fully compliant with the guidelines of the National Health and Medical Research Council ethical standards (HREC 931149X, 1034765, 110525, 1339373, 010411, 1647448, and 1750632) and was carried out in accordance with the Declaration of Helsinki. The patients/participants provided their written informed consent to participate in this study.

## Author Contributions

CS contributed to the conception and design of the study and protocol. CS, LD and SC participated in the assessment and clinical classifications. RP-C, CS and LD provided scientific input into the paper. RP-C and CS performed the statistical analysis plan and statistical analysis, interpreted the results, and wrote and edited the manuscript. All authors have read and approved the final version of the manuscript and agreed with the order of presentation of the authors.

## Conflict of Interest

The authors declare that the research was conducted in the absence of any commercial or financial relationships that could be construed as a potential conflict of interest.

## Supplementary Material

Supplementary Figure 1 Flow chart of participants in the study.

Supplementary Figure 2 Mean standardized uptake value ratios (SUVRs) according to physical activity (PA) tertiles and APOE-ε4 genotypes.

## References

[B1] Abellan van KanG.RollandY.AndrieuS.BauerJ.BeauchetO.BonnefoyM.. (2009). Gait speed at usual pace as a predictor of adverse outcomes in community-dwelling older people an International Academy on Nutrition and Aging (IANA) task force. J. Nutr. Health Aging 13, 881–889. 10.1007/s12603-009-0246-z19924348

[B2] Arena R. Myers J. Williams M. A. Gulati M. Kligfield P. Balady G. J.. (2007). Assessment of functional capacity in clinical and research settings: a scientific statement from the american heart association committee on exercise, rehabilitation, and prevention of the council on clinical cardiology and the council on cardiovascular nursing. Circulation 116, 329–343. 10.1161/CIRCULATIONAHA.106.184461 17576872

[B3] Bohannon R. W. (2006). Reference values for the timed up and go test: a descriptive meta-analysis. J. Geriatr. Phys. Ther. 29, 64–68. 10.1519/00139143-200608000-00004 16914068

[B4] Brown B. M. Peiffer J. J. Taddei K. Lui J. K. Laws S. M. Gupta V. B.. (2013). physical activity and amyloid-beta plasma and brain levels: results from the Australian imaging, biomarkers and lifestyle study of ageing. Mol. Psychiatry 1875–1881. 10.1038/mp.2012.107 22889922

[B5] Bull F. C. Al-Ansari S. S. Biddle S. Borodulin K. Buman M. P. Cardon G.. (2020). World health organization 2020 guidelines on physical activity and sedentary behaviour. Br. J. Sports Med. 54, 1451–1462. 10.1136/bjsports-2020-102955 33239350 PMC7719906

[B6] Caspersen C. J. Powell K. E. Christenson G. M. (1985). Physical activity, exercise, and physical fitness: definitions and distinctions for health-related research. Public Health Rep. 100, 126–131. 3920711 PMC1424733

[B7] Ciarmiello A. Giovannini E. Riondato M. Giovacchini G. Duce V. Ferrando O.. (2019). Longitudinal cognitive decline in mild cognitive impairment subjects with early amyloid-beta neocortical deposition. Eur. J. Nucl. Med. Mol. Imaging 46, 2090–2098. 10.1007/s00259-019-04409-1 31264171

[B8] Craig C. L. Marshall A. L. Sjostrom M. Bauman A. E. Booth M. L. Ainsworth B. E.. (2003). International physical activity questionnaire: 12-country reliability and validity. Med. Sci. Sports Exerc. 35, 1381–1395. 10.1249/01.MSS.0000078924.61453.FB 12900694

[B9] Dao E. Hsiung G. R. Sossi V. Tam R. Shahinfard E. Nicklin E.. (2019). Cerebral amyloid-beta deposition is associated with impaired gait speed and lower extremity function. J Alzheimers Dis. 71, S41–S49. 10.3233/JAD-180848 30741682

[B10] de Souto Barreto P. Andrieu S. Payoux P. Demougeot L. Rolland Y. Vellas B.. (2015). Physical activity and amyloid-beta brain levels in elderly adults with intact cognition and mild cognitive impairment. J. Am. Geriatr. Soc. 63, 1634–1639. 10.1111/jgs.13530 26200930

[B11] Duara R. Loewenstein D. A. Lizarraga G. Adjouadi M. Barker W. W. Greig-Custo M. T.. (2019). Effect of age, ethnicity, sex, cognitive status and APOE genotype on amyloid load and the threshold for amyloid positivity. Neuroimage Clin. 22:101800. 10.1016/j.nicl.2019.101800 30991618 PMC6447735

[B12] Ekelund U. Dalene K. E. Tarp J. Lee I. M. (2020). Physical activity and mortality: what is the dose response and how big is the effect? Br. J. Sports Med. 54, 1125–1126. 10.1136/bjsports-2019-101765 31964630

[B13] Erickson K. I. Leckie R. L. Weinstein A. M. (2014). Physical activity, fitness, and gray matter volume. Neurobiol. Aging 35, S20–S28. 10.1016/j.neurobiolaging.2014.03.034 24952993 PMC4094356

[B14] Folstein M. F. Folstein S. E. McHugh P. R. (1975). “Mini-mental state”. a practical method for grading the cognitive state of patients for the clinician. J. Psychiatr. Res. 12, 189–198. 10.1016/0022-3956(75)90026-6 1202204

[B15] Frederiksen K. S. Gjerum L. Waldemar G. Hasselbalch S. G. (2019). Physical activity as a moderator of alzheimer pathology: a systematic review of observational studies. Curr. Alzheimer Res. 16, 362–378. 10.2174/1567205016666190315095151 30873924

[B16] Gandy S. (2005). The role of cerebral amyloid beta accumulation in common forms of Alzheimer disease. J. Clin. Invest. 115, 1121–1129. 10.1172/JCI25100 15864339 PMC1087184

[B17] Head D. Bugg J. M. Goate A. M. Fagan A. M. Mintun M. A. Benzinger T.. (2012). Exercise engagement as a moderator of the effects of APOE genotype on amyloid deposition. Arch. Neurol. 69, 636–643. 10.1001/archneurol.2011.845 22232206 PMC3583203

[B18] Kannel W. B. McGee D. Gordon T. (1976). A general cardiovascular risk profile: the framingham study. Am. J. Cardiol. 38, 46–51. 10.1016/0002-9149(76)90061-8 132862

[B19] Kim J. Y. Oh D. Sung K. Choi H. Paeng J. C. Cheon G. J.. (2020). Visual interpretation of [(18)F]Florbetaben PET supported by deep learning-based estimation of amyloid burden. Eur J. Nucl. Med. Mol. Imaging 48, 1116–1123. 10.1007/s00259-020-05044-x 32990807

[B20] Kyu H. H. Bachman V. F. Alexander L. T. Mumford J. E. Afshin A. Estep K.. (2016). Physical activity and risk of breast cancer, colon cancer, diabetes, ischemic heart disease, and ischemic stroke events: systematic review and dose-response meta-analysis for the global burden of disease study 2013. BMJ 354:i3857. 10.1136/bmj.i3857 27510511 PMC4979358

[B21] Landau S. M. Marks S. M. Mormino E. C. Rabinovici G. D. Oh H. O'Neil J. P.. (2012). Association of lifetime cognitive engagement and low beta-amyloid deposition. Arch. Neurol. 69, 623–629. 10.1001/archneurol.2011.2748 22271235 PMC3747737

[B22] Lawton M. P. Brody E. M. (1969). Assessment of older people: self-maintaining and instrumental activities of daily living. Gerontologist 9, 179–186. 10.1093/geront/9.3_Part_1.179 5349366

[B23] Liang K. Y. Mintun M. A. Fagan A. M. Goate A. M. Bugg J. M. Holtzman D. M.. (2010). Exercise and Alzheimer's disease biomarkers in cognitively normal older adults. Ann. Neurol. 68, 311–318. 10.1002/ana.22096 20818789 PMC2936720

[B24] Livingston G. Huntley J. Sommerlad A. Ames D. Ballard C. Banerjee S.. (2020). Dementia prevention, intervention, and care: 2020 report of the lancet commission. Lancet 396, 413–446. 10.1016/S0140-6736(20)30367-6 32738937 PMC7392084

[B25] Long J. Cai T. Huang X. Zhou Y. Kuang J. Wu L. (2019). Reference value for the TUGT in healthy older people: a systematic review and meta-analysis. Geriatr. Nurs. 41, 325–330. 10.1016/j.gerinurse.2019.11.012 31810729

[B26] Nadkarni N. K. Perera S. Snitz B. E. Mathis C. A. Price J. Williamson J. D.. (2017). Association of brain amyloid-beta with slow gait in elderly individuals without dementia: influence of cognition and apolipoprotein E epsilon4 genotype. JAMA Neurol. 74, 82–90. 10.1001/jamaneurol.2016.3474 27842173 PMC5735996

[B27] Nelson M. E. Rejeski W. J. Blair S. N. Duncan P. W. Judge J. O. King A. C.. (2007). Physical activity and public health in older adults: recommendation from the american college of sports medicine and the american heart association. Circulation 116, 1094–1105. 10.1161/CIRCULATIONAHA.107.185650 17671236

[B28] Okonkwo O. C. Schultz S. A. Oh J. M. Larson J. Edwards D. Cook D.. (2014). Physical activity attenuates age-related biomarker alterations in preclinical AD. Neurology 83, 1753–1760. 10.1212/WNL.0000000000000964 25298312 PMC4239838

[B29] Perani D. Schillaci O. Padovani A. Nobili F. M. Iaccarino L. Della Rosa P. A.. (2014). A survey of FDG- and amyloid-PET imaging in dementia and GRADE analysis. Biomed. Res. Int. 2014, 1–1. 10.1155/2014/246586 24772437 PMC3977528

[B30] Podsiadlo D. Richardson S. (1991). The timed “Up & Go”: a test of basic functional mobility for frail elderly persons. J. Am. Geriatr. Soc. 39, 142–148. 10.1111/j.1532-5415.1991.tb01616.x 1991946

[B31] Rabin J. S. Klein H. Kirn D. R. Schultz A. P. Yang H. S. Hampton O.. (2019). Associations of physical activity and beta-amyloid with longitudinal cognition and neurodegeneration in clinically normal older adults. JAMA Neurol. 76:1203. 10.1001/jamaneurol.2019.1879 31312836 PMC6635892

[B32] Rowe C. C. Ellis K. A. Rimajova M. Bourgeat P. Pike K. E. Jones G.. (2010). Amyloid imaging results from the australian imaging, biomarkers and lifestyle (AIBL) study of aging. Neurobiol. Aging 31, 1275–1283. 10.1016/j.neurobiolaging.2010.04.007 20472326

[B33] Schultz S. A. Boots E. A. Almeida R. P. Oh J. M. Einerson J. Korcarz C. E.. (2015). Cardiorespiratory fitness attenuates the influence of amyloid on cognition. J. Int. Neuropsychol. Soc. 21, 841–850. 10.1017/S1355617715000843 26581795 PMC4716656

[B34] Szoeke C. Coulson M. Campbell S. Dennerstein L. Gatewa P. (2016). Cohort profile: women's healthy ageing project (WHAP) - a longitudinal prospective study of Australian women since 1990. Womens Midlife Health 2:5. 10.1186/s40695-016-0018-y 30766701 PMC6300017

[B35] Szoeke C. Goodwill A. M. Gorelik A. Dennerstein L. Caeyenberghs K. Simpson S.. (2019). Apolipoprotein E4 mediates the association between midlife dyslipidemia and cerebral amyloid in aging women. J. Alzheimers Dis. 68, 105–114. 10.3233/JAD-180815 30689578

[B36] Tian Q. Resnick S. M. Bilgel M. Wong D. F. Ferrucci L. Studenski S. A. (2017). Beta-amyloid burden predicts lower extremity performance decline in cognitively unimpaired older adults. J. Gerontol. A. Biol. Sci. Med. Sci. 72, 716–723. 10.1093/gerona/glw183 27664990 PMC6075426

[B37] Valenzuela P. L. Castillo-Garcia A. Morales J. S. de la Villa P. Hampel H. Emanuele E.. (2020). Exercise benefits on Alzheimer's disease: state-of-the-science. Ageing Res. Rev. 62:101108. 10.1016/j.arr.2020.101108 32561386

[B38] Vemuri P. Lesnick T. G. Przybelski S. A. Knopman D. S. Roberts R. O. Lowe V. J.. (2012). Effect of lifestyle activities on Alzheimer disease biomarkers and cognition. Ann. Neurol. 72, 730–738. 10.1002/ana.23665 23280791 PMC3539211

[B39] Villemagne V. L. Ong K. Mulligan R. S. Holl G. Pejoska S. Jones G.. (2011). Amyloid imaging with (18)F-florbetaben in Alzheimer disease and other dementias. J. Nucl. Med. 52, 1210–1217. 10.2967/jnumed.111.089730 21764791

[B40] Wennberg A. M. V. Savica R. Hagen C. E. Roberts R. O. Knopman D. S. Hollman J. H.. (2017). Cerebral amyloid deposition is associated with gait parameters in the mayo clinic study of aging. J. Am. Geriatr. Soc. 65, 792–799. 10.1111/jgs.14670 27869301 PMC5397339

[B41] WHO (2019). Risk Reduction of Cognitive Decline And Dementia: WHO Guidelines. Geneva: World Health Organization. 31219687

[B42] Wirth M. Haase C. M. Villeneuve S. Vogel J. Jagust W. J. (2014). Neuroprotective pathways: lifestyle activity, brain pathology, and cognition in cognitively normal older adults. Neurobiol. Aging 35, 1873–1882 10.1016/j.neurobiolaging.2014.02.015 24656834 PMC4019766

