# Supplementary figures and images for: Influence of Physical Activity Levels and Functional Capacity on Brain β-Amyloid Deposition in Older Women
Source: Front Aging Neurosci. 2021 Jul 9;13:697528. doi: 10.3389/fnagi.2021.697528 (PMC8300898; doi:10.3389/fnagi.2021.697528)

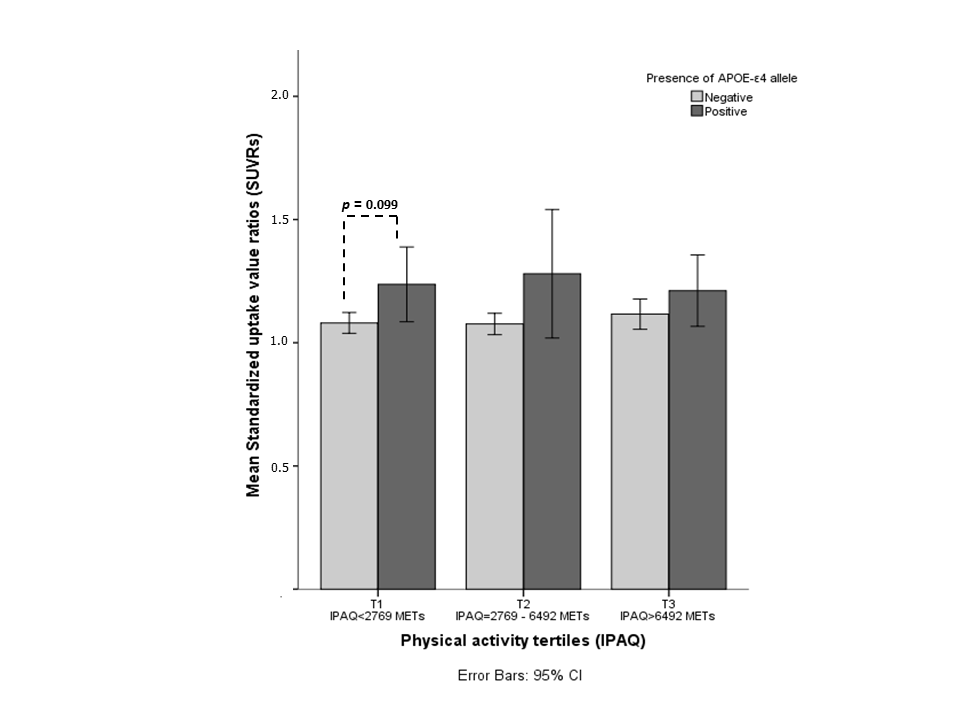

Supplement: Supplementary Figure 1 — Flow chart of participants in the study. [file Data_Sheet_1.ZIP › Supplementary Figure 2.tif]

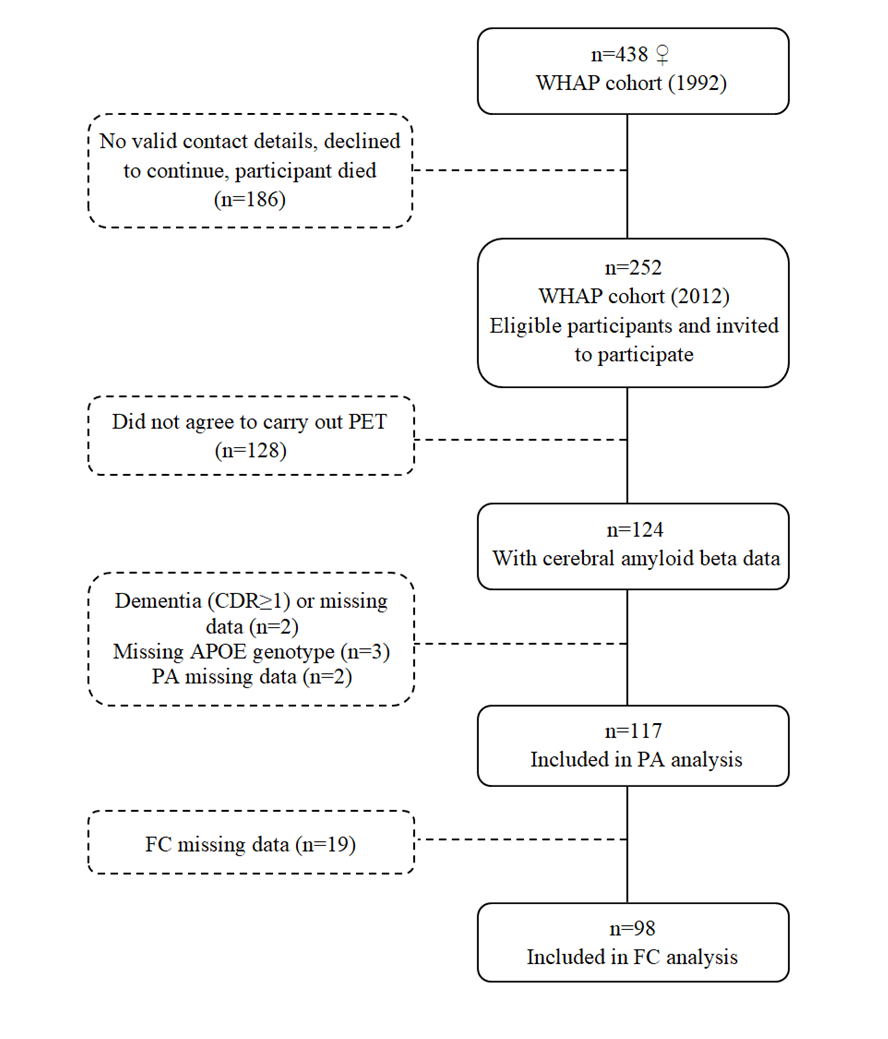

Supplement: Supplementary Figure 1 — Flow chart of participants in the study. [file Data_Sheet_1.ZIP › Supplementary Figure 1.tif]
